# Psychosocial work experience after implementing hybrid work: a longitudinal study

**DOI:** 10.1186/s12889-026-27487-x

**Published:** 2026-04-24

**Authors:** Caroline Corneliuson, Maral Babapour Chafi, Andreas Tornevi, Albin Stjernbrandt, Viktoria Wahlström

**Affiliations:** 1 Department of Epidemiology and Global Health, Umeå, Sweden; 2 The Institute of Stress Medicine, Region Västra Götaland, Gothenburg, Sweden; 3 Division Design & Human Factors, Chalmers University of Technology, Gothenburg, Sweden

**Keywords:** Telework, Occupational health, Office workers, Psychosocial work environment, Remote work

## Abstract

**Background:**

Hybrid work has become a prevalent work model, referring to the combination of office and remote work. Previous studies have found that hybrid work arrangements may positively affect psychosocial factors such as job satisfaction, work-life balance, and retention. However, hybrid work has also been observed to blur the boundaries between work and private life and increase work-family conflict, and more knowledge is needed on how hybrid work influences the psychosocial work environment from a longitudinal perspective. This study aimed to investigate the impact of the transition to hybrid work on the work experience, and to determine how personal and occupational factors, such as age, sex, managerial position, number of children living at home, work tasks, autonomy, and time spent working at the office, influenced the change in work experience.

**Methods:**

To explore differences in the Work Experience Measurement Scale (WEMS), longitudinal questionnaire data (*n* = 148) from white-collar workers in a medium-sized Swedish municipality were analyzed. Data collection took place in 2017 before COVID-19 and 2023, 1.5 years after national work-from-home restrictions were lifted. Univariate linear regression was used to determine how the different factors affected the change in work experience.

**Results:**

The mean age of the study population was 50.1 years at the time of the second data collection in 2023. The study populations’ WEMS scores significantly improved over time, both on the total scale and specifically within the dimensions supportive working conditions, time experience, autonomy, and leadership. The main factor that influenced the work experience was sex, where women’s work experience improved significantly over time, whereas men’s work experience did not.

**Conclusion:**

This study contributes a longitudinal perspective on hybrid work and its impact on the psychosocial work environment. While the findings of this study align with previous research in relation to improvement of, for example, job satisfaction, the study also reveals sex differences previously not seen in a hybrid work context.

## Background

 Hybrid work has quickly become a commonly used term as well as a prevalent work model; it refers to the combination of remote and office work with communication via digital tools and face-to-face encounters [1]. The COVID-19 pandemic facilitated a quick shift towards remote work, increasing the share of European remote workers from about 3% of knowledge workers pre-pandemic [2] to approximately 30% of men and 40% of women working from home in 2021 [3]. However, the prevalence of remote work varies substantially across the continent, and hybrid work is more prevalent in Northern European countries compared to countries in Eastern and Southern Europe [3]. Reflecting this regional trend, about 46% of the Swedish working population engaged in remote work to some extent in 2024 [4].

The psychosocial work environment is challenging to define as it encompasses many diverse aspects, but it often refers to features in the work organization and/or personal factors that affect employees and may be explored at the micro-, meso-, and macro-level [5]. Research on the topic often focuses on how psychosocial factors such as job demands, work organization, job content, or social relations at work affect different outcomes, for example, health and illnesses [5].

Previous research has shown that remote work could enhance employees’ work-life balance, increase perceived autonomy and work control, and improve job satisfaction [6, 7]. The increased autonomy, with the flexibility to choose to work remotely, seems to have positive effects on the psychosocial work environment [8]. Research suggests that many employees prefer to work remotely between one and three days per week, but those exceeding their preferred amount of remote work report poorer well-being, highlighting the importance of autonomy in choosing remote workdays for maintaining satisfaction and well-being [9, 10]. Additionally, the possibility of hybrid work seems to increase the intention to stay within an organization, making retention a key benefit for employers [11, 12]. Thus, it is of interest to further explore how autonomy and the amount of time spent working remotely affect the work experience. One persistent challenge brought up in previous studies is difficulties in separating work from personal life; working from home seems to lead to longer working hours, thus blurring boundaries and hindering recovery [6, 13]. Blurred boundaries between work and family life have also been seen to lead to more work-family conflict [8, 14, 15]. Having children living at home seems to be a possible factor associated with increased work-family conflict [15]. Family constellations, sex, and age are therefore relevant personal factors to explore in relation to perceived work experience.

Even though hybrid work means employees regularly perform work remotely, the office remains an essential space for coworker interaction, offering spontaneous meetings and social interactions that strengthen professional relationships [16]. In flexible office environments such as activity-based offices, tasks that require concentration may be negatively affected [17]. Employees who have high-concentration individual tasks may perceive activity-based offices to fit their needs poorly [18]. Furthermore, non-individual tasks, where employees collaborate, seem to fit poorly with an open-space office, due to a lack of control over one’s social interaction and communication with others [18, 19]. A previous study showed that managers did not experience the same decline in productivity as employees following relocation to activity-based offices, likely due to differences in work tasks and job characteristics [17]. Furthermore, research indicates that leadership faces new challenges in hybrid settings, with managers facing increased workload, greater coordination demands, and difficulties leading teams remotely [7, 16]. Thus, perceptions of hybrid work may differ between managers and employees. These previous findings suggest that the nature of one’s work tasks and work content could play a role in shaping how employees and managers perceive their work experience in a hybrid work model. Previous studies have mainly relied on cross-sectional data, resulting in a significant gap in understanding hybrid work from a longitudinal perspective [8, 20]. Furthermore, despite growing insights into the immediate impacts of hybrid work, significant knowledge gaps remain concerning the effects on the psychosocial work environment [6, 8]. Traditionally, research on the psychosocial work environment has focused on risk factors, but adopting a salutogenic approach could offer the possibility to focus on positive health factors [21]. To guide the development of future work environments, further knowledge is needed on how hybrid work interacts with task types, office designs, and employee autonomy.

The first aim of our study was to investigate the longitudinal impact of the transition to hybrid work on the work experience. The second aim was to determine how autonomy, amount of time spent working remotely, personal factors, and work content influenced the change in the work experience.

## Method

### Design and setting

In this longitudinal study, we studied changes in psychosocial work experience over time. Baseline data were collected via a self-report survey in 2017 from white-collar workers in a medium-sized Swedish municipality (approximately 56,000 inhabitants), followed up by a survey in October–November of 2023, approximately one and a half years after the COVID-19 pandemic restrictions had been lifted in Sweden.

Between the years 2014–2017, the organization had transformed most of its cell-based offices into activity-based offices [17], which meant that the organization, to some extent, committed to a flexible way of working even before the COVID-19 pandemic. During the pandemic, the studied organization enforced remote work due to restrictions imposed by Swedish governmental authorities. The restrictions meant that everyone who could work from home was requested to do so. When implementing remote work during the pandemic, the employer provided computer screens, input devices, and office chairs for the remote workstations at the employees’ request. After the pandemic, a hybrid work model was implemented for all white-collar workers as part of the municipality’s ongoing efforts to become a workplace of the future. The implemented hybrid work model gave the employees varying opportunities to work from home based on their roles, teams, and managers.

The baseline survey was sent to 512 office workers, and 407 responses were received, with a total response rate of 80%. The follow-up survey was sent to 554 office workers, and 378 responded, yielding a response rate of 68%. Two reminder emails were sent for each data collection period. Out of the survey responders, a total of 148 people responded to the survey both at baseline and follow-up. A dropout analysis was performed to assess potential attrition bias.

### Background variables

The employees’ autonomy to decide their work location, we used the question “How much freedom of choice do you have to decide to what extent you work from home?” with response alternatives: “I have full freedom of choice to what I work from home”, “I have some freedom of choice to what extent I work from home”, and “I have no freedom of choice to what extent I work from home”. the amount of time spent working at the office was assessed with the question: “I work at the office:” with the response alternatives: 1–3 times a month, 1 day a week, 2–3 days a week, and 4–5 days a week.

Personal factors, including data on sex and age were collected at both baseline and follow-up. The presence of children in the household was assessed by the question: “Do you have children living at home?”

To assess the work content, we used the question: ”Does your work demand you to…“1) discuss with colleagues several times a day”, “2) be fully concentrated”, and “3) work individually on administrative tasks”, using the response alternatives: never, rarely, sometimes, often, and always. Office type was assessed with the question: “What office type best describes your current office space when you are at the office?” Where the office types to choose from were: cell-office, shared room (2–3 people), open-plan office, and no assigned workplace, as defined by [22]. Before analyses, responses were dichotomized into having a permanent desk or not.

### Outcome variables

The Swedish version of the Work Experience Measurement Scale (WEMS) was used to assess the work experience [21]. WEMS was originally developed to provide an understanding of the work experience from a salutogenic perspective and has been tested and shown adequate reliability [21, 23]. WEMS has also been used in studies to explore work experiences in office settings across the health care, social work, and private sectors in Sweden [24, 25].

WEMS comprises 32 items and is divided into six dimensions that, in total, reflect a salutogenic perspective on the work experience among respondents [21]. Each dimension includes between three and seven claims each, focusing on their respective topics: supportive working conditions, internal work experience, autonomy, time experience, leadership, and process of change [21].

The claims were assessed on a six-level scale from “do not agree” to “fully agree”. Each response is assigned a score, which is combined into a total index score for the whole scale as well as for each individual dimension. The total index score can be standardized into a scale between 0 and 100, with higher scores indicating a more positive work experience [21].

### Data processing and statistical analyses

The data processing was performed in two steps. Firstly, we calculated a total index score for each respondent based on the WEMS scale and its six dimensions. We then standardized the scores on a scale from 0 to 100 to enable comparison across respondents. T-tests for paired samples were then used to compare the mean scores between the different time points. For the second aim, the change in scores between the baseline and follow-up was calculated, both for the total WEMS score and for the six separate dimensions, by subtracting the baseline score from the follow-up score. These variables were then utilized to explore potential explanatory factors between the time points using linear regression models. Additional analyses of each item and their development over time were also performed, see Table 4 in Appendix.

Univariate linear regression models were conducted, with the WEMS change scores as the dependent variables. Models were constructed using the total score. Univariate models were constructed for each factor respectively. The factors were: sex, age, managerial position, amount of remote work, autonomy in choosing amount of remote work, office type, and having children living at home. These factors were selected a priori based on the authors’ pre-understanding of the psychosocial work environment and what might affect it.

Before analyzing the work tasks as factors, the response categories were collapsed to obtain more balanced group sizes. For the questions relating to work tasks, such as discussing with co-workers and needing full concentration, the answers were dichotomized into rarely/sometimes and often/always. The answers for the administrative tasks were grouped into never, rarely/sometimes, and often/always.

All analyses were conducted with SPSS (version 29.0.1 for Windows, IBM Corporation, Armonk, NY, USA). Statistical significance was set at *p* < 0.05.

## Results

### Background characteristics

Out of the 148 respondents, a total of 117 (79%) were female, the mean age was 50 (SD 9.1), and 25% held a managerial position at follow-up. The majority of the study population worked in an activity-based office environment, as reflected in the 35% of the employees who had an assigned office space/desk. About half (53%) of the employees spent 2–3 days a week at the office (Table 1).

**Table 1:** Background characteristics of the study group from the follow- up in 2023 (*n* = 148)

	*N* (%)	Mean (SD)
Degree of autonomy to choose work location (*n* = 145)		
Full autonomy	50 (34.5)	
Some autonomy	91 (62.8)	
No autonomy	4 (2.8)	
Time spent at the office (*n* = 127)		
1–3 times/month	9 (7.1)	
1 time/week	15 (11.8)	
2–3 times/week	67 (52.8)	
4–5 times/week	36 (28.3)	
Personal factors		
* Age* (*n* = 147)		50.1 (9.1)
* Sex* (*n* = 148)		
Male	31 (20.9)	
Female	117 (79.1)	
* Children living at home* (*n* = 147)		
Yes	80 (54.4)	
No	67 (45.6)	
Work content		
* Managerial position* (*n* = 146)		
Yes	34 (23.1)	
No	113 (76.9)	
* Permanent office space* (*n* = 140)		
Yes	50 (35.7)	
No	90 (64.3)	
* Work tasks discussed with colleagues several times a day* (*n* = 146)		
Never	3 (2.1)	
Rarely	8 (5.5)	
Sometimes	43 (29.5)	
Often	76 (52.1)	
Always	16 (11.0)	
* Individual work tasks requiring concentration* (*n* = 146)		
Never	-	
Rarely	2 (1.4)	
Sometimes	16 (11.0)	
Often	100 (68.5)	
Always	28 (19.2)	
* Individual administrative tasks* (*n* = 146)		
Never	5 (3.4)	
Rarely	18 (12.3)	
Sometimes	32 (21.9)	
Often	76 (52.1)	
Always	15 (10.3)	

### Work experience measures over time

We found an increase in the mean score of the total WEMS scale (*p* = 0.008), as well as for the dimensions: supportive working conditions (*p* = 0.021), autonomy (*p* = 0.005), time experience (*p* < 0.001), and leadership (*p* = 0.039) (Table 2).

**Table 2:** Changes in the standardized index of WEMS between baseline and follow-up (*n* = 148)

	Baseline	Follow-up	95% CI	*p*-value
	Mean	Mean		Effect
WEMS total score (*n* = 122)	71.2	74.3	0.8–5.4	0.008
Supportive working conditions (*n* = 128)	77.9	80.9	0.4–5.3	0.021
Internal work experience (*n* = 130)	81.1	83.5	−0.2-5.0	0.067
Autonomy (*n* = 129)	63.6	68.0	1.5–7.4	0.005
Time experience (*n* = 131)	48.5	55.7	3.1–11.5	< 0.001
Leadership (*n* = 131)	75.8	79.3	0.2–6.9	0.039
Process of change (*n* = 130)	63.8	65.5	−3.3–6.8	0.498

When analyzing individual items within each dimension, time experience was the only dimension in which all items showed a significant increase (Supplementary Table 1). For the other dimensions, only a few items contributed to driving the change for the whole dimension. For example, within the leadership dimension, only one of the six items significantly increased: “My manager is available when I need him/her” (*p* < 0.001).

### Factors of influence on change in work experience

For the univariate linear regression models, 122 respondents who had completed the entire instrument were included. The regression models showed that several factors were associated with increased WEMS scores over time, indicating a more positive work experience. Sex was associated with changes in work experience, where women experienced an improved work experience over time with 4.3 points (95% CI 1.7–6.8), whereas men did not. This was also the only factor where the difference between the factor levels was significant (*p* = 0.05) (Table 3).

For the other factors, no statistically significant differences were observed between the factor levels. Managers’ work experience stayed the same over time and non-managers had an increased WEMS score, but the difference between the factor levels was not statistically significant (*p* = 0.17). Factors related to having children living at home, work tasks, having an assigned office space/desk, level of freedom to choose work locations, or time spent at the office were not significantly associated with changes in WEMS scores (Table 3).

**Table 3:** The change in total indexed WEMS scores between baseline and follow-up analyzed using univariate linear regressions. (*n* = 122)

	Change (95% CI)	*p*-value	*R*^2^ (%)
Degree of autonomy to choose work location			5.8
No freedom (*n* = 3)	1.7 (− 12.9–16.3)	Ref.	
Some freedom (*n* = 75)	3.4 (0.5–6.3)	0.82	
Full freedom (*n* = 44)	2.7 (− 1.1–6.5)	0.89	
Amount of time spent working at the office			6.4
1–3 days per month (*n* = 8)	8.4 (− 0.5–17.3)	Ref.	
1 day a week (*n* = 14)	3.5 (− 3.3–10.2)	0.38	
2–3 days a week (*n* = 59)	2.5 (− 0.7–5.8)	0.22	
4–5 days a week (*n* = 29)	1.4 (− 3.3–6.0)	0.17	
Personal factors			
*Age* (*n* = 122)	0.04 (− 0.2–0.3)		1
*Sex*			8.7
Female (*n* = 96)	4.3 (1.7–6.8)	Ref.	
Male (*n* = 26)	-1.2 (− 6.1–3.7)	0.05	
*Children living at home*			6
Yes (*n* = 66)	2.4 (− 0.7–5.5)	Ref.	
No (*n* = 56)	3.9 (0.5–7.2)	0.53	
Work content			
*Managerial position*			7.2
Yes (*n* = 28)	0.2 (− 4.5–4.9)	Ref.	
No (*n* = 94)	4.0 (1.4–6.5)	0.17	
*Permanent office place/desk*			7.1
Yes (*n* = 45)	5.1 (1.3–8.8)	Ref.	
No (*n* = 77)	2.0 (− 0.9–4.8)	0.19	
*Work tasks discussed with colleagues*			7.8
Never/Rarely/sometimes (*n* = 44)	5.6 (1.8–9.3)	Ref.	
Often/always (*n* = 78)	1.7 (− 1.1–4.5)	0.16	
*Individual work tasks requiring concentration*			7.1
Never/Rarely/sometimes (*n* = 15)	7.2 (0.7–13.6)	Ref.	
Often/always (*n* = 107)	2.5 (0.1–5.0)	0.19	
*Individual administrative tasks*			6.5
Never/Rarely/sometimes (*n* = 47)	4.5 (0.9–8.1)	Ref.	
Often/Always (*n* = 75)	2.2 (− 0.7–5.1)	0.33	

The dropout analysis revealed no significant difference in sex or managerial position between the study group and the dropout group. The dropout group was 4.3 years older than the study group (*p* < 0.001). The total WEMS score at baseline was 3.5 points lower for the dropout group than the study group (*p* = 0.02).

## Discussion

This study utilized a longitudinal approach to investigate the impacts of the transition to hybrid work on the psychosocial work experience. Even though the organization studied had high scores before the pandemic, indicating a healthy work environment, the work experience further improved significantly after the transition to hybrid work.

A statistically significant increase was identified in the following dimensions: supportive working conditions, autonomy, time experience, and leadership, implying that these dimensions were positively affected by the transition to hybrid work. Previous cross-sectional studies have found that psychosocial factors such as job satisfaction and perceived autonomy improve during hybrid work [6, 7]. This is in line with our findings, where job satisfaction (“Feeling happy about one’s job”) contributed to the increase in the dimension for supportive working conditions. The item “My manager is available when I need him/her” contributed to the increase in the leadership dimension. Research conducted during the pandemic also indicates improvements in employees’ relationships with their managers, possibly due to many organizations promoting regular check-ins [6].

The only dimension of WEMS where all items showed a statistically significant improvement was time experience. The items in this dimension capture workers’ ability to finish work on time, without stress, and within regular working hours. Our study offers new insight into how time is experienced during hybrid work, and to the best of our knowledge, there are no other studies on this topic. Previous research has shown an increase in perceived working hours [15], and a Swedish report also found an increase in self-reported working hours during hybrid work [4]. Our study further contributes to previous research showing that hybrid work can improve not only job satisfaction but also employee well-being, as well as increasing loyalty with lower turnover rates without compromising productivity [9–11]. Taken together these findings finding indicate that offering flexible work solutions is beneficial to both employees and employers.

Sex was the only factor with a significant difference between the factor levels, where women’s work experience improved after hybrid work was introduced, while men’s remained unchanged. There are very few studies on sex differences within a hybrid work context. One previous report indicates that women telework more often [3], and the authors discuss that gendered divisions of labor, both in the workplace and at home, may explain this pattern. Previous studies with samples and contexts that differ from our current study have found that women might be better at virtual communication, and that women’s psychological safety increases while working hybrid [26, 27]. In Swedish public sector organizations, such as the municipality studied in our current study, it has been shown that gender inequalities persist, where men are usually found in higher positions and in places with better working conditions [28]. However, within female-dominated organizations, issues such as higher job demands and fewer resources are usually experienced similarly by men and women [29]. This could suggest, there may be underlying conditions within the context of hybrid work that benefit women and their work experience more than men, in the current study, that we did not consider in our analysis.

Interestingly, we found that the managers’ work experience remained unchanged after the introduction of hybrid work. Previous qualitative studies have found that hybrid work has led managers to reassess their roles, expanding their experienced duties to also include managing well-being, as well as negotiating terms for remote work [30]. Furthermore, research before, during and after the COVID-19 pandemic indicates that while managers’ workload increased with remote work, they also found that managers had more challenges and experienced difficulties in leading remotely, as it was described as requiring more effort [7, 16, 31]. Similar challenges have also been identified in research on flexible offices, that require leading distributed work groups [32–34]. However, WEMS does not effectively capture underlying challenges related to hybrid leadership in comparison with, for example, qualitative interviews or questionnaires focusing specifically on managers’ tasks and their specific work environments. Speculatively, there may be aspects such as organizational structures, ways of working, or guidelines within the studied organization that alleviate difficulties observed in other studies and that have not been accounted for in our analysis.

Previous studies have indicated that the amount of time spent working remotely may affect job satisfaction and performance [35], and highlighted that autonomy in choosing remote workdays can be a mediator for maintaining well-being among workers [8, 10]. However, our study found that neither the amount of time working remotely nor the amount of freedom to choose were explanatory factors for the change in WEMS score. While the findings of the current study do not indicate that these factors are explanatory, it is possible that our study did not capture mismatches between actual and preferred remote work, which have been reported [10].

### Strengths and limitations

A strength of this study is its longitudinal design combined with the possibility to examine several factors that may impact the work experience in a hybrid work context. A limitation, however, is the relatively small study population. A potential recruitment bias could also be considered, as individuals with particularly positive or negative experiences may have been more likely to participate.

Future studies, preferably with larger longitudinal sample size, are needed to further investigate the effects of hybrid work. As an example of a possible limitation related to statistical power, the current study did not find managerial position to be an explanatory factor for changes in work experience over time, even though managers did not increase their WEMS score in the same manner as employees. It is, however, important to further study managers’ experiences in hybrid work settings, as this topic remains under-researched. Moreover, future research should also consider that hybrid work may be enacted in diverse ways. Different combinations of work locations, work settings, and job demands may form distinct patterns of hybrid work that may be associated with different changes of psychosocial work outcomes over time. Identifying such variation would help clarify for whom and under what conditions hybrid work is most beneficial.

While the results of the current study indicate positive outcomes for hybrid work, it is possible that they may be explained by factors this study was unable to control for. The use of WEMS as a measure of the psychosocial work environment provided a salutogenic perspective often lacking in other measures that tend to focus on a pathogenic perspective and preventing harm [21, 36]. On the other hand, we did not consider risk factors or challenges, for example, in relation to managers’ experiences. The generalizability of our findings is somewhat limited and likely applies primarily to similar contexts in Swedish, Nordic, and European settings. In general, future studies should continue examining the longitudinal effects of hybrid work on the psychosocial work environment, since it is still a relatively new phenomenon, and we are still only able to understand a limited number of aspects of long-term effects for employees and employers.

## Conclusions

In summary, the current study found that the work experience improved after the transition to hybrid work, especially among women. However, future studies should investigate sex and gender differences further to develop a deeper understanding of which factors might be beneficial for women and men n a hybrid work model. The results of our study add to existing research by highlighting that hybrid work offers positive effects for both employees and employers, and could be a beneficial solution for arranging office work.

## Data Availability

Access to data can be provided for research purposes but is restricted by laws regarding the privacy of research participants and therefore not made publicly available.

## Appendix

**Table 4:** Changes in the scores of WEMS items between baseline and follow-up (*n* = 148)

	Baseline	Follow-up	*p*-value
Supportive working conditions			
We encourage and support each other at work	5.24	5.12	0.165
There is a good atmosphere where I work	5.10	5.19	0.258
I think the work routines function well	4.35	4.98	< 0.001
I get feedback on the work I do	4.52	4.73	0.023
I am happy about my job	4.98	5.22	0.023
I feel that my employer invests in my health	4.75	4.78	0.870
I get advice and practical help from others when necessary	5.30	5.27	0.696
Internal work experience			
I feel that my work is meaningful	5.40	5.47	0.247
I feel that my work situation makes me grow	5.20	5.07	0.195
There is variety in my work	5.39	5.43	0.654
I do the work I was trained for	5.04	5.12	0.402
I am happy when I go to work	4.89	4.96	0.456
My work is a great personal challenge	4.37	5.02	< 0.001
Autonomy			
I decide when to do the various work tasks	4.38	4.58	0.038
I decide what to do in my work	3.93	4.05	0.274
I decide how to do my work	4.56	4.69	0.173
I decide my own work pace	3.85	4.28	< 0.001
Time experience			
I have enough time during my normal working hours to do my job without time pressure (stress)	3.50	3.90	0.002
I always have time to finish each work task in the way it is supposed to be done	2.73	3.01	0.05
I do not need to work more than my scheduled hours	4.04	4.44	0.003
Leadership			
My boss is available when I need him/her	4.76	5.18	< 0.001
My boss is good at making us interested and committed to our work	4.55	4.64	0.477
My boss helps us divide our work in a fair way	4.43	4.55	0.313
My boss takes things up for discussion in my work group before making important decisions	4.67	4.82	0.223
When necessary, my boss is able to make his/her own decisions	5.19	5.28	0.405
My boss makes sure that information on the operation’s goals and visions is available to my work group	5.16	5.33	0.107
Process of Change			
The process of change was done with an open dialogue	4.36	4.43	0.629
The process of change was a result of the needs and wishes of my work group	3.88	3.95	0.694
The process of change was important to me	4.25	4.31	0.678
We all had an active part in the process of change	4.11	4.28	0.269
I felt safe in my work with regard to the process of	4.25	4.33	0.594
I was well informed about the process of change	4.28	4.37	0.550

## Abbreviations

WEMS: Work Experience Measurement Scale

## References

[1] Lauring J, Jonasson C. What is hybrid work? Towards greater conceptual clarity of a common term and understanding its consequences. Hum Resour Manag Rev. 2025;35(1):101044.

[2] Eurofund. Teleworkability and the COVID-19 crisis: a new digital divide? Publications Office of the European Union, editor. Luxembourg: Publications Office of the European Union; 2020. Availble from: https://www.eurofound.europa.eu/en/publications/all/teleworkability-and-covid-19-crisis-new-digital-divide.

[3] European Commission: Directorate-General for Justice and Consumers, Flexible working arrangements and gender equality in Europe. Publications Office of the European Union. 2024. Available from: 10.2838/13215.

[4] Statistiska centralbyrån. Labour Force Surveys (LFS) 2025:1 - Theme: Work from home and hours worked. Örebro: Statistiska centralbyrån. 2025. Available from: https://www.scb.se/hitta-statistik/statistik-efter-amne/arbetsmarknad/utbud-av-arbetskraft/arbetskraftsundersokningarna-aku/pong/publikationer/arbetskraftsundersokningarna-aku-20251--tema-arbete-hemifran-och-arbetad-tid/ English summary available from: https://www.scb.se/publication/54902.

[5] Rugulies R. What is a psychosocial work environment? Scand J Work Environ Health. 2019;45(1):1–6. 10.5271/sjweh.3792 30643919

[6] Gajendran RS, Ponnapalli AR, Wang C, Javalagi AA. A dual pathway model of remote work intensity: A meta-analysis of its simultaneous positive and negative effects. Pers Psychol. 2024;77(4):1351–86.

[7] Ipsen C, van Veldhoven M, Kirchner K, Hansen JP. Six Key advantages and disadvantages of working from home in Europe during COVID-19. Int J Environ Res Public Health. 2021;18(4):1826. 10.3390/ijerph18041826 PMC7917590 33668505

[8] Vleeshouwers J, Fløvik L, Christensen JO, Johannessen HA, Bakke Finne L, Mohr B, et al. The relationship between telework from home and the psychosocial work environment: a systematic review. Int Arch Occup Environ Health. 2022;95(10):2025–51. 35829741 10.1007/s00420-022-01901-4 PMC9652268

[9] Barrero JM, Bloom N, Davis S. Why working from home will stick. 2021. Available from: 10.3386/w28731.

[10] Heiden M, Hallman DM, Svensson M, Mathiassen SE, Svensson S, Bergström G. Mismatch between actual and preferred extent of telework: cross-sectional and prospective associations with well-being and burnout. BMC Public Health. 2023;23(1):1736. 37674141 10.1186/s12889-023-16683-8 PMC10481552

[11] Bloom N, Han R, Liang J. Hybrid working from home improves retention without damaging performance. Nature. 2024;630(8018):920–5. 38867040 10.1038/s41586-024-07500-2 PMC11208135

[12] Mutiganda JC, Wiitavaara B, Heiden M, Svensson S, Fagerström A, Bergström G et al. A systematic review of the research on telework and organizational economic performance indicators. Front Psychol. 2022;13:1035310. 10.3389/fpsyg.2022.1035310 PMC9812566 36619046

[13] Fonner KL, Stache LC. All in a day’s work, at home: teleworkers’ management of micro role transitions and the work–home boundary. New Technol Work Employ. 2012;27(3):242–57.

[14] Sun M, Kraus T, Pauli R, Garus C. Changing sense of place in hybrid work environments: A systematic review of place identity and employee well-being. Wellbeing Space Soc. 2025;8:100236.

[15] Antunes ED, Bridi LRT, Santos M, Fischer FM. Part-time or full-time teleworking? A systematic review of the psychosocial risk factors of telework from home. Front Psychol. 2023;14 :1065593. 10.3389/fpsyg.2023.1065593 PMC9992198 36910835

[16] Babapour Chafi M, Hultberg A, Bozic Yams N. Post-Pandemic Office Work: Perceived Challenges and Opportunities for a Sustainable Work Environment. Sustainability. 2021;14(1):294.

[17] Öhrn M, Wahlström V, Harder MS, Nordin M, Pettersson-Strömbäck A, Bodin Danielsson C et al. Productivity, satisfaction, work environment and health after relocation to an activity-based flex office: the active office design study. Int J Environ Res Public Health. 2021;18(14):7640. 10.3390/ijerph18147640 PMC8304243 34300090

[18] Hoendervanger JG, Van Yperen NW, Mobach MP, Albers CJ. Perceived Fit and User Behavior in Activity-Based Work Environments. Environ Behav. 2022;54(1):143–69.

[19] Haapakangas A, Hallman DM, Mathiassen SE, Jahncke H. The effects of moving into an activity-based office on communication, social relations and work demands – A controlled intervention with repeated follow-up. J Environ Psychol. 2019;66:101341.

[20] Ropponen A. Remote work – the new normal needs more research. Scand J Work Environ Health. 2025;2:53–7. 10.5271/sjweh.4213 PMC11886879 39868483

[21] Nilsson P. Development and quality analysis of the Work Experience Measurement Scale (WEMS). Work. 2010;35(2):153–61. 20164610 10.3233/WOR-2010-0967

[22] Bodin Danielsson C, The Office - An Explorative Study. : Architectural Design’s Impact on Health, Job Satisfaction & Well-being [Internet] [PhD Dissertation]. Stockholm: KTH; 2010. Available from: https://urn.kb.se/resolve?urn=urn:nbn:se:kth:diva-24429.

[23] Nilsson P, Andersson HI, Ejlertsson G. The Work Experience Measurement Scale (WEMS): A useful tool in workplace health promotion. WORK. 2013;45(3):379–87. 23324702 10.3233/WOR-121541

[24] Larsson I, Ahlstrand I, Larsson M, Pennbrant S, Ekman A, Hallgren J. Health-promoting resources and workplace experiences among newly graduated healthcare and social work professionals – a multicentre cross-sectional study. BMC Health Serv Res. 2025;25(1). 10.1186/s12913-025-12782-x PMC12039133 40301847

[25] Bergström J, Miller M, Horneij E. Work environment perceptions following relocation to open-plan offices: A twelve-month longitudinal study. WORK. 2015;50(2):221–8. 24284691 10.3233/WOR-131798

[26] Hill NS, Hincapie MX. Gender differences in virtual collaboration effectiveness in hybrid teams. Inf Manag. 2025;62(3):104107.

[27] Back H, Back P, Kriuchkov I. Leveling the Playing Field? Effects of Hybrid Work on the Psychological Safety of Minority Groups. In: Proceedings of the 58th Hawaii International Conference on System Sciences (HICSS). 2025 Jan 7–10; Waikoloa Village, HI, USA. pp. 6639–48.

[28] Welander J, Lornudd C, Schwarz U, Göransson S. Navigating Gender Inequalities in Working Conditions: Accountable Politicians’ Perspectives on Their Work Environment Responsibility in Swedish Municipal Organizations. Scand J Public Adm. 2025;29(1):1.

[29] Nyberg A, Härenstam A, Johansson G, Peristera P. Psychosocial Working Conditions for Women and Men in Industries with Different Types of Production and Gender Composition: Sweden, 1991–2017. In: Keisu B-I, Tafvelin S, Brodin H, editors. Gendered Norms at Work: New Perspectives on Work Environment and Health. Cham: Springer International Publishing; 2021. pp. 35–61.

[30] Hasle M, Manca C, Edwards K, Ipsen C. It’s not a journey I booked the ticket for: unfolding line manager sensemaking in hybrid work. Int J Workplace Health Manag. 2025;18(3):280–96.

[31] Edvardsson IR, Gardarsdottir J. Navigating Uncharted Waters: Exploring Leaders’ Challenges in the Era of COVID-19 and the Rise of Telework. Sustainability. 2023;15(23):16471.

[32] Babapour Chafi M, Nordin M, Wahlström V, Pettersson-Strömbäck A. Navigating productivity dilemmas and conflicting loyalties in activity-based flexible offices - A qualitative study of managers’ perspectives and coping strategies. PLoS ONE. 2025;20(11):e0335945. 41270118 10.1371/journal.pone.0335945 PMC12637956

[33] Hansson J, Vinberg S, Wall E, Löfstrand P. The transition to an activity-based workplace: Experiences of managers and employees from a sense of coherence perspective in public sector workplaces. PLoS ONE. 2025;20(3):e0320324. 40163423 10.1371/journal.pone.0320324 PMC11957360

[34] Vinberg S, Ejnebrand R, Löfstrand P. New forms of leadership in activity-based work environments in combination with working from home—A qualitative study of managers in Swedish public sector workplaces. Front Organizational Psychol. 2026;3. 10.3389/forgp.2025.1699712.

[35] Rudolph CW, Zacher H. Working from home: When is it too much of a good thing? Hum. Resour Dev Q. 2025;36(1):9–47.

[36] Nebbs A, Martin A, Neil A, Dawkins S, Roydhouse J. An integrated approach to workplace mental health: a scoping review of instruments that can assist organizations with implementation. Int J Environ Res Public Health. 2023;20(2):1192. 36673948 10.3390/ijerph20021192 PMC9859114

